# Supplementary material for: The Self-Identification Program (SIP): A Clinically Implemented Third-Wave CBT Deepening Dysfunctional Self-Identification in Mood Disorders
Source: Medicina (Kaunas). 2025 Nov 20;61(11):2071. doi: 10.3390/medicina61112071 (PMC12654058; doi:10.3390/medicina61112071)
Supplement: Supplementary file 1 [file medicina-61-02071-s001.zip › medicina-3972785-supplementary.pdf]

Supplementary File S1. Self-Identification Program (SIP): Session Structure (Clinical Outline)

SIP-1 — Eight-Session Protocol

Essence and goals:
The first phase of SIP aims to help participants:

1. Recognize the scope of their mind and its natural powers.
2. Use these powers as the foundation for more correct self-identification.
3. Prepare for SIP-2, which deepens this training.

Each session (2 hours each) introduces one "Power of Mind", practiced experientially and consolidated through daily application.

1) A Pacifying Power

Patients will learn to:

- Stop identifying with a self limited by restlessness or dependence on external satisfaction.
- Identify with the natural peace of mind discovered through mindful breathing.
- When agitation arises, use breathing to restore calm and ease the drive for constant stimulation.

2) A Reassuring Power

Patients will learn to:

- Stop identifying with a self limited by feelings of damage or fear of introspection.
- Identify with the stable clarity of awareness that remains unharmed despite painful experiences.
- When insecurity appears, reconnect with this inner clarity to re-establish confidence and safety.

3) A Beneficial Power

Patients will learn to:

- Stop identifying with a self limited by feelings of unworthiness.
- Identify with the unceasing value of a kind and caring heart.
- When self-criticism arises, recall one’s good heart to reduce dependence on external reassurance.

4) A Discerning Power

Patients will learn to:

- Stop identifying with a self limited by confusion or lack of awareness.
- Identify with the natural purity and discernment of the mind, supported by kindness.
- Reaffirm the capacity to notice thoughts and emotions clearly without judgment.

5) A Prioritizing Power

Patients will learn to:

- Stop identifying with a self limited by intolerance of strong emotions.
- Identify with the joyful, stable mind that can regulate emotions through non-reactivity.
- When anger or frustration arises, practice pausing and allowing emotion to settle before acting.

6) An Acting Power

Patients will learn to:

- Stop identifying with a self limited by indecision or uncertainty about what matters.
- Identify with a compassionate and active self guided by meaningful intentions.
- Remember that others share similar struggles; act from clarity and warmth rather than avoidance.

7) A Creative Power

Patients will learn to:

- Stop identifying with a self limited by feelings of inadequacy or uselessness.
- Identify with the creative potential that transforms everyday events into opportunities for learning and growth.
- Recognize daily experiences as training grounds for developing beneficial qualities.

8) A Liberating Power

Patients will learn to:

- Stop identifying with a self limited by trauma, guilt, or the belief of being "too damaged."
- Identify with the wise, joyful mind that can transform suffering into understanding.

- When the “traumatized self” arises, recognize it as a story and reconnect with calm, clarity, and warmth.

SIP-2 — Twenty-One-Session Protocol

Essence & goals:
Building on the “powers of the mind” recognized in SIP-1, SIP-2 deepens this through three levels of scope: *Initial, Intermediate,* and *Great*.
Each session (2 hours each) invites patients to stop identifying with a limiting self and to cultivate a corresponding correct self-identification.

I. Initial Scope

1. Our Precious Human Life

Patients will learn to:

- Stop identifying with a self limited by lacking good fortune.
- Identify with an unlimited beneficial potential empowered by awareness of present, highly fortunate opportunities for realizing correct self-identification.

2. Death and Impermanence

Patients will learn to:

- Stop identifying with a self limited by being too indolent, busy, and distracted.
- Identify with an unlimited beneficial potential empowered by awareness of death and impermanence—the possible imminent cessation of these fortunate opportunities.

3. The Danger of the Inner Paths to Traumatization

Patients will learn to:

- Stop identifying with a self limited by dependence on attachment, aversion, and indifference.
- Identify with an unlimited beneficial potential empowered by awareness that these three tendencies are inner paths to being traumatized again.

4. Sources of Support (Refuge, Secularized)

Patients will learn to:

- Stop identifying with a self limited by feeling unsupported.
- Identify with an unlimited beneficial potential empowered by awareness of three valid sources of support:
    1. The inspiration of therapists who have advance in correct self-identification.
    2. The therapeutic method of correct self-identification itself.

3. The help of those sincerely applying correct self-identification.

5. Actions and Their Effects

Patients will learn to:

- Stop identifying with a self limited by having no control over experiences.
- Identify with an unlimited beneficial potential empowered by awareness that experiences arise from one's intentions, and therefore train in restraining actions motivated by attachment, aversion, or indifference.

II. Intermediate Scope

6. Developing Renunciation for Incorrect Self-Identification

Patients will learn to:

- Stop identifying with a self limited by dissatisfaction with past experiences and present conditions.
- Identify with an unlimited beneficial potential empowered by awareness that as long as we identify incorrectly, we can only live in frustration—thus incorrect identification is an ultimate object of renunciation.

III. Great Scope

7. Developing Equanimity

Patients will learn to:

- Stop identifying with a self limited by dependence on how one feels treated by others.
- Identify with an unlimited beneficial potential empowered by awareness that one can maintain a warm attitude toward all beings, independent of others' behavior.

8. Recognizing the Kindness of Caregivers

Patients will learn to:

- Stop identifying with a self limited by conceptions of caregivers who failed them.
- Identify with an unlimited beneficial potential empowered by recognizing caregivers' contributions to present fortunate conditions for correct self-identification.

9. Remembering the Kindness of All Living Beings

Patients will learn to:

- Stop identifying with a self limited by living in a negative world.
- Identify with an unlimited beneficial potential empowered by awareness that we are interconnected in a web of kindness from which it is impossible to separate ourselves.

10. Equalizing Self and Others

Patients will learn to:

- Stop identifying with a self limited by a self-centered view of the world.
- Identify with an unlimited beneficial potential empowered by awareness that all beings equally wish to be happy and free from suffering.

11. The Disadvantages of Self-Cherishing

Patients will learn to:

- Stop identifying with a self limited by believing "I am most important" while neglecting others.
- Identify with an unlimited beneficial potential empowered by awareness that problems arise from cherishing an incorrect self-identification.

12. The Advantages of Cherishing Others

Patients will learn to:

- Stop identifying with a self limited by the idea that cherishing others is dangerous.
- Identify with an unlimited beneficial potential empowered by awareness that positive qualities come from cherishing the beneficial potential of all beings.

13. Exchanging Self with Others

Patients will learn to:

- Stop identifying with a self limited by belief in its own natural supremacy.
- Identify with an unlimited beneficial potential empowered by awareness of the benefits of viewing oneself as "other" and others as "I."

14. Great Compassion

Patients will learn to:

- Stop identifying with a self limited by feeling threatened by others' suffering.
- Identify with an unlimited beneficial potential empowered by awareness that universal compassion is part of one's true nature.

15. Taking

Patients will learn to:

- Stop identifying with a self limited by avoiding suffering at all costs.
- Identify with an unlimited beneficial potential empowered by recognizing that one can reduce the causes of one's problems (self-cherishing) by mentally taking on the suffering and delusions of others.

16. Wishing Love

Patients will learn to:

- Stop identifying with a self limited by inability to take responsibility for others' happiness.
- Identify with an unlimited beneficial potential empowered by awareness that promoting others' happiness is a source of the greatest joy.

17. Giving

Patients will learn to:

- Stop identifying with a self limited by lack of resources.
- Identify with an unlimited beneficial potential empowered by awareness that wishing to give freely fosters a sense of abundance and unceasing joy.

18. Wishing to Identify Correctly for the Benefit of All

Patients will learn to:

- Stop identifying with a self limited by having too many faults.
- Identify with an unlimited beneficial potential empowered by awareness that training in virtue and beneficial action is a path to realizing one's full potential.

19. Tranquil Abiding

Patients will learn to:

- Stop identifying with a self limited by lack of control over the mind.
- Identify with an unlimited beneficial potential empowered by awareness that a supple, stable mind arises from training in concentration.

20. Superior Seeing

Patients will learn to:

- Stop identifying with a self limited by perceiving the world and self as fixed or inherently existent.
- Identify with an unlimited beneficial potential empowered by awareness that all phenomena are interdependent and lack fixed essence.

21. Developing an Inner Guide

Patients will learn to:

- Stop identifying with a self limited by its perceived inability to change.
- Identify with an unlimited beneficial potential by exchanging the habitual view of self for a view informed by the understanding and skills developed in this program with the therapist.
